# Measurement of Symptom Change Following Web-Based Psychotherapy: Statistical Characteristics and Analytical Methods for Measuring and Interpreting Change

**DOI:** 10.2196/10200

**Published:** 2018-07-12

**Authors:** Eyal Karin, Blake F Dear, Gillian Z Heller, Milena Gandy, Nickolai Titov

**Affiliations:** ^1^ eCentreClinic Department of Psychology Macquarie University Sydney Australia; ^2^ Mindspot Clinic Macquarie University Sydney Australia; ^3^ Department of Statistics Faculty of Science and Engineering Macquarie University Sydney Australia

**Keywords:** clinical measurement, treatment evaluation, symptom change, symptom scales, psychotherapeutic change

## Abstract

**Background:**

Accurate measurement of treatment-related change is a key part of psychotherapy research and the investigation of treatment efficacy. For this reason, the ability to measure change with accurate and valid methods is critical for psychotherapy.

**Objective:**

The aims of this study were to (1) explore the underlying characteristics of depressive symptom change, measured with the nine-item Patient Health Questionnaire (PHQ-9), following psychotherapy, and (2) compare the suitability of different ways to measure and interpret symptom change. A treatment sample of Web-based psychotherapy participants (n=1098) and a waitlist sample (n=96) were used to (1) explore the statistical characteristics of depressive symptom change, and (2) compare the suitability of two common types of change functions: linear and proportional change.

**Methods:**

These objectives were explored using hypotheses that tested (1) the relationship between baseline symptoms and the rate of change, (2) the shape of symptom score distribution following treatment, and (3) measurement error associated with linear and proportional measurement models.

**Results:**

Findings demonstrated that (1) individuals with severe depressive baseline symptoms had greater reductions in symptom scores than individuals with mild baseline symptoms (11.4 vs 3.7); however, as a percentage measurement, change remained similar across individuals with mild, moderate, or severe baseline symptoms (50%-55%); (2) positive skewness was observed in PHQ-9 score distributions following treatment; and (3) models that measured symptom change as a proportional function resulted in greater model fit and reduced measurement error (<30%).

**Conclusions:**

This study suggests that symptom scales, sharing an implicit feature of score bounding, are associated with a proportional function of change. Selecting statistics that overlook this proportional change (eg, Cohen *d*) is problematic and leads to (1) artificially increased estimates of change with higher baseline symptoms, (2) increased measurement error, and (3) confounded estimates of treatment efficacy and clinical change. Implications, limitations, and idiosyncrasies from these results are discussed.

## Introduction

Accurate measurement of treatment-related change is a key part of psychotherapy research [1-3] and the investigation of treatment efficacy [4-6]. For example, measurable change in symptoms of anxiety and depression is often used as the primary means to research and test the safety of emerging treatments [7]. Reporting symptom change in anxiety and depression has been shown to describe the clinical trajectory of participants in treatment [8], illustrate the cost-effectiveness of treatment [9], and compare treatments [10]. For this reason, the ability to measure change with accurate and valid methods is critical for psychotherapy [6,11].

Several statistical and clinical methods are employed to increase the validity and accuracy of change measurement in psychotherapy. The most common methodology in psychotherapy research is the combined use of standardized scales, such as standardized symptom scales of anxiety [12] or depression [1,13], and the use of statistical analyses, such as Cohen *d* effect sizes, that measure and interpret the rate of change in treatment [4-6]. Many types of standardized scales are available for measuring and interpreting change in treatment (eg, clinical interviews, measurement of behavior or quality of life [14]), and that change can be statistically estimated through various statistical methods [15]. However, from the wide range of possible methods for measuring treatment outcomes [16], the use of standardized scales, primarily symptom scales, in combination with effect sizes, primarily Cohen *d*, are the most influential. For example, symptom scales and effect sizes are used to evaluate treatment-related change and treatment efficacy within psychotherapy trials [17-19], epidemiological studies [20,21], meta-analytic studies of various treatments [22], and are even mandated within clinical guidelines for reporting in clinical trials, such as Consolidated Standards of Reporting Trials (CONSORT) [19], Transparent Reporting of Evaluations with Nonrandomized Designs (TREND) [23], Strengthening the Reporting of Observational studies in Epidemiology (STROBE) [24], and others [11].

Notwithstanding the common use of both symptom scales and effect sizes for measuring psychotherapeutic-related change, little research is currently available to verify or refute the use of different statistical methods for measuring and interpreting symptom change [25,26]. For example, the use of effect sizes, such as Cohen *d*, is based on statistical assumptions that change is linear. In technical terms, by employing effect sizes, researchers assume that the symptom change that follows treatment is average, constant, and representative of the average change experienced by any participating individual [18,27]. Put another way, if an average individual with moderate depressive symptoms prior to treatment, such as a score between 10 and 15 on the nine-item Patient Health Questionnaire (PHQ-9), would improve by 5 points on a symptom scale, an individual with severe baseline symptoms (eg, PHQ-9 score of 20-27) would be expected to demonstrate the same rate of improvement (eg, 5 points). Similarly, under the linear assumption, a group of participants with different baseline symptoms (eg, mild, moderate, or severe baseline symptoms) undertaking the same therapy would be expected to have similar effect sizes between groups (eg, 1.0). However, in contrast to the common use of statistics that assume change is linear, there are two lines of research to suggest that real-world symptom change may occur as a proportional function from baseline. First, psychological treatment studies often describe an increased rate of clinical change within samples of increased baseline symptom severity [20,28]. Second, common symptom scales, such as the PHQ-9 [29], the Generalized Anxiety Disorder seven-item scale (GAD-7) [30], and prominent others (eg, Kessler Psychological Distress scale) [31], often demonstrate an implicit design feature of score bounding at minimal symptoms. This bounding within symptom scales should theoretically imply that, under effective treatment, all individuals would reduce their symptoms down to the same endpoint of minimal levels [1,9] and that the rate of change would systematically depend on an individual’s symptoms at baseline [32,33].

From a statistical point of view, identifying the characteristics of symptom change, and employing a suitable statistical analysis that captures the underlying function of change, can fundamentally impact both the measurement and interpretation of clinical outcomes [15,34,35]. For example, under circumstances in which change is proportional in nature, the selection of a proportional statistical analysis can greatly increase the accuracy and validity of estimating longitudinal clinical change [34,35]; the detection of moderators of symptom change [36]; the classification of subgroups, such as remitters or nonresponders [37]; as well as the ability to research other objectives [38]. For this reason, the function of symptom change must be researched and more clearly understood. Such research could verify, refute, and draw out the implication for using well-established statistical methods (eg, effect sizes, linear statistics) and emerging alternatives (eg, percentage improvement, generalized linear statistics) for measuring and interpreting change in treatment. In addition, researching the function and characteristics of symptom change has the potential to inform researchers and the broader community about the type of change individuals in treatment are likely to experience.

### This Study

This study aims to (1) explore the fundamental statistical characteristics of treatment-related depressive symptom change and (2) compare the implications from measuring and interpreting clinical change through effect sizes, such as Cohen *d*, against emerging alternatives, such as percentage improvement (proportional, generalized longitudinal linear statistics) [25,26].

This study employed a large sample of individuals (N=1098) who underwent Web-based psychotherapy (Internet-delivered cognitive behavioral therapy [ICBT]) [39] for symptoms of depression (PHQ-9 [29]). Although Web-based psychotherapy represents a distinct type of psychotherapy, the use of Web-based treatments, which standardizes treatment materials and participant engagement through automatization, can be seen as an opportunity for researching symptom change with high internal validity and minimum methodological interference.

The statistical characteristics of symptom change were explored with three steps. Initially, the relationship between baseline symptoms and the rate of change was explored. In line with previous clinical studies that suggest that more severely symptomatic participants demonstrate increased effect sizes [20,32], it was hypothesized that individuals with increased symptoms at baseline would also demonstrate increased rates of symptom change (hypothesis 1). Second, the shape of symptom score distribution before and following treatment were explored. In line with the suggestion that symptoms scores are bounded at minimal symptoms [29,30], the distributions of pretreatment and posttreatment depression symptom levels were hypothesized to show evidence of positive skewness and kurtosis at both pretreatment and posttreatment (hypothesis 2). Third, the measurement error associated with linear and proportional measurement models was compared. In line with the characterization of symptom change as proportional, it was hypothesized that those statistical methods that measure symptom change as a proportional function would be associated with reduced measurement error and indicate greater statistical fit to real symptom data in treatment (hypothesis 3). Finally, an additional effort was taken to explore the patterns of depressive symptom change within a control group (n=96). This addition was designed to explore the pattern of symptom change that is not specific to treatment.

## Methods

### The Sample

This study combined clinical data from three published randomized controlled trials, all of which evaluated ICBT for symptoms of depression and anxiety [39,40]. These interventions were almost identical in structure and therapeutic content. All trials were delivered using the same evidence-based online treatment approach [7] and were conducted within the same research clinic, the eCentreClinic [41]. A precautionary test, aiming to compare the symptom reduction rates between the individual trials, demonstrated similarities across all three interventions. Specifically, a generalized estimated equation (GEE) model [35], testing the longitudinal symptom change of each trial, resulted in slight differences in the estimates of symptom change across trials (PHQ-9 range 5.23-6.29 points); differences were not statistically significant (group × time: Wald χ^2^_2,2368_=5.0, *P*=.08).

Together, these trials represent a large random intake of self-selecting adults into treatment over a period of 2 years with a total of 1262 adult participants, of whom 1098 (87.01%) were successfully assessed at both pretreatment and posttreatment time points. Additional information about recruitment, advertising, treatment materials, and additional treatment procedures can be found within additional eCentreClinic publications [7,41].

To be included in these trials, participants were selected on the basis of (1) demonstrating at least mild symptoms of depression or anxiety (a minimum score ≥5 on either the PHQ-9 or the GAD-7), (2) older than 18 years and younger than 65 years, (3) being an Australian resident, and (4) having Internet access for the period of the trial. In addition, applicants who reported a score of 3 (considered severe) on item 9 of the PHQ-9 measuring suicidal risk, were referred to another service.

Additional demographic and symptom characteristics are shown in Table 1 for both the treatment and waitlist control conditions.

**Table 1:** Sample demographics (N=1194).

Demographics		Collated treatment sample (n=1098)	Control sample (n=96)
Gender (male), n (%)		330 (30.1)	51 (53.1)
Age (years), mean (SD)		52.8 (14.2)	56.3 (13.0)
Using medication during the course, n (%)		351 (31.9)	51 (53.1)
Married, n (%)		713 (64.9)	45 (46.9)
Employed, n (%)		636 (57.9)	49 (51.0)
**Education, n (%)**			
	High school	176 (16.0)	39 (40.6)
	Vocational education	307 (27.9)	24 (25.0)
	Degree	615 (56.0)	37 (38.5)
**PHQ-9^a^, mean (SD)**			
	Before treatment	11.73 (4.83)	10.95 (4.73)
	following treatment)	5.60 (4.58)	11.00 (5.04)
**GAD-7^b^, mean (SD)**			
	Before treatment	10.91 (4.53)	9.5 (4.53)
	Following treatment	5.47 (4.35)	8.83 (4.67)

^a^PHQ-9: nine-item Patient Health Questionnaire..

^b^GAD-7: seven-item Generalized Anxiety Disorder scale.

### Symptom Measure

The PHQ-9 was employed as the primary outcome variable, measuring the presence and severity of depressive symptoms [29]. The PHQ-9 is widely used in clinical trials [7,16], comprising nine items, with high internal consistency and high sensitivity to the presence and change of clinical depression diagnoses [29]. Scores on the PHQ-9 correspond to the cumulative experience of common depressive symptoms over the preceding 2-week period. Cumulative scores range from 0 to 27 and scores are clinically interpreted as falling within five categories: (1) no depression symptoms (total score: 0-4), (2) mild depression symptoms (total score: 5-9), (3) moderate depression symptoms (total score: 10-14), (4) moderately severe depression symptoms (total score: 15-19), and (5) very severe depression symptoms (total scores: 20-27). Symptom scores were modified with a small constant added (0.001) to ensure that plausible values of zero symptoms at posttreatment were represented in the model when statistically modeling proportional functions, such as logarithmic link functions.

### Analytical Plan

The function of symptom change was explored with three separate steps, corresponding to the three hypotheses.

The first hypothesis that individuals with increased symptoms at baseline would also demonstrate increased rates of symptom change was tested by examining the relationship between baseline symptoms and the rate of symptom change. Symptom change was examined within the five subgroups of individuals of different baseline PHQ-9 score bands (eg, minimal to no symptoms to very severe depression symptoms). Within each subgroup, the rate of change was approximated with GEE models, multilevel models [34], and raw means. These methods represent common longitudinal statistical methods in clinical trials [42]. The estimation of change through all three GEE, mixed models, and raw scores was designed to clarify that the underlying function of symptom change could be identified when using various statistical models.

Under a linear pattern of symptom change, participants of any baseline symptoms would be expected to show a similar rate of improvement overall. That is, an average symptom change score that would be observed across individuals, irrespective of the severity of their symptoms at baseline [18]. In contrast, under a proportional pattern of symptom change, participants presenting with increased baseline symptom severity would likely show larger symptom change compared to those individuals with mild or moderate baseline symptoms [15].

To test the second hypothesis that distributions of pretreatment and posttreatment depression symptom levels would show evidence of positive skewness and kurtosis, the distributions of depression symptoms scores at both pretreatment and posttreatment were evaluated for evidence of skewness. In this step, if the dataset would present with statistically normal distribution of symptom scores at both time points, the symptom change over time would be considered as linear. In contrast, if symptoms changed as a proportional function from baseline, positive skewness should be observed, particularly at posttreatment, where individuals from various baseline symptoms would shift and concentrate around the symptom score band of minimal symptoms. Graphical and numerical explorations of pre-post score distributions were included.

To test the third hypothesis that statistical methods measuring symptom change as a proportional function would be associated with reduced measurement error and indicate greater statistical fit to real symptom data in treatment, the relative measurement accuracy of models that represent either linear or proportional symptom change were compared. Specifically, this step compared model fit statistics and the remaining unexplained (residual) variance associated with each function of change. Both mixed models and GEE models were run initially as models that assume change was linear, represented through models that specified a normal scale of the dependent variable and an identify link function. Following this, alternative statistical models were compared, which specified a gamma scale and a log link function; representing models that assumed change was proportional. Generally, the gamma scale is considered a suitable method for data showing signs of skewness and multiplicative change function [15]; however, the selection of the gamma scale does not imply that alternative multiplicative statistical methods (eg, negative binomial scale, Poisson scale, or zero inflated models) would be less effective.

**Figure 1:**
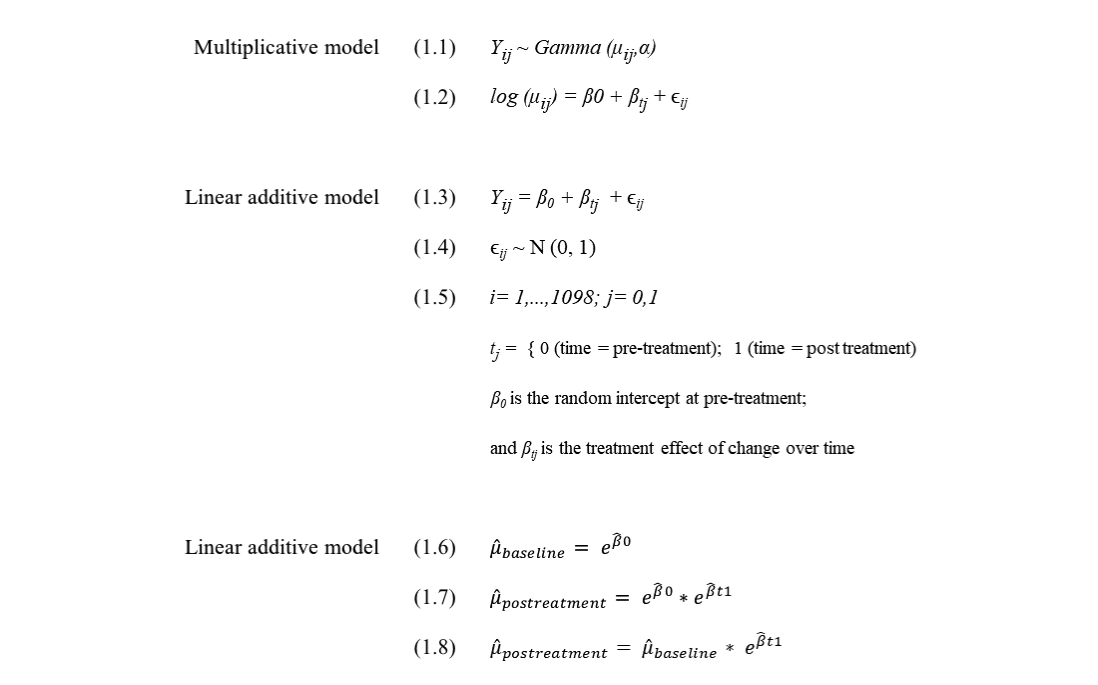
Equations 1.1-1.8.

Formulas emphasizing the difference in statistical notation between the multiplicative model (Equations 1.1-1.2) and the linear model (Equations 1.3-1.5) are presented in Figure 1. With more formal statistical notation, the multiplicative effect within the log link model is created when the intercept, *β*_0_, or baseline symptoms, is multiplied by the treatment effect, *β*_tj_, the estimate of exponential change following treatment (Equations 1.6-1.8 in Figure 1).

The suitability of either model type was evaluated through model fit statistics, generated using SAS 9.4 software. Specifically, the quasilikelihood under the independence model criterion (QIC) statistic [43] for GEE models, and Akaike information criterion (AIC) and Bayesian information criterion (BIC) for mixed effects models [44], compared between linear (additive) and generalized linear (proportional) models. Within all AIC, BIC, and QIC model fit estimates, relatively lower scores imply overall reduced variance, and overall increase measurement accuracy.

In addition to model fit statistics, the measurement error associated with the assumption that symptom change was either a fixed average score, or a percentage improvement score, was compared. In this step, measurement error was created for each participant by comparing the predicted posttreatment score under each change assumption (eg, PHQ-9 change of 5 points or 50% from baseline) against a known participant outcome score at posttreatment. The difference between the expected symptom outcome and actual treatment outcome effectively represents measurement error under the two change assumptions, akin to residual scores and measurement error variance. The pattern of residuals created under either assumption of symptom change was explored in two ways. First, the total quantity of error variance under each function was compared. Second, measurement residuals were graphically explored under each function of symptom change by comparing the increase or decrease of residuals for individuals with different baseline symptom score.

## Results

**Figure 2:**
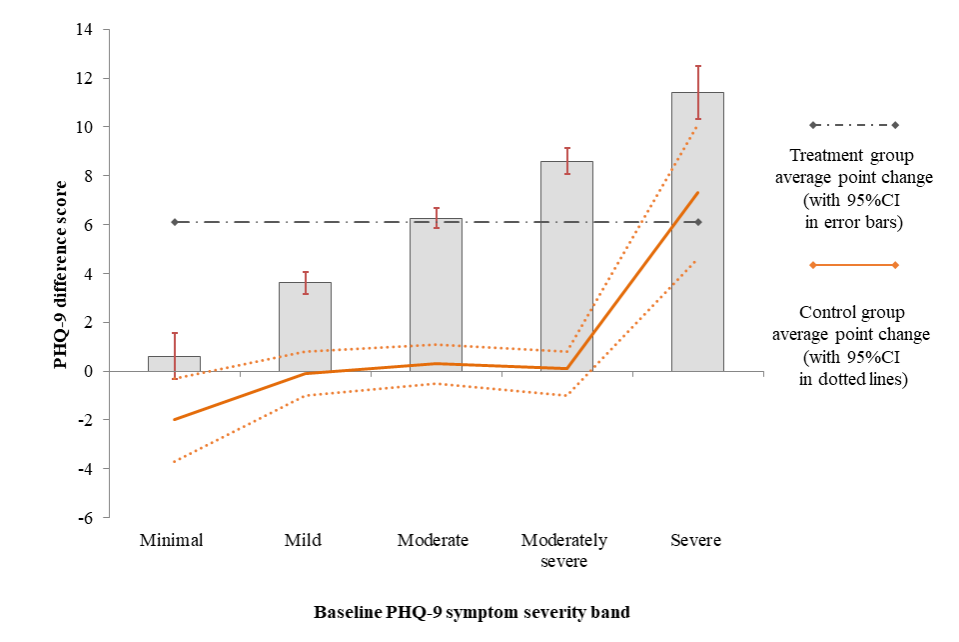
Measurement of mean treatment-related PHQ-9 symptom change per initial pretreatment symptom severity band; whiskers represent 95% CI s. Symptom change observed under control conditions indicated by a solid trend line.

**Figure 3:**
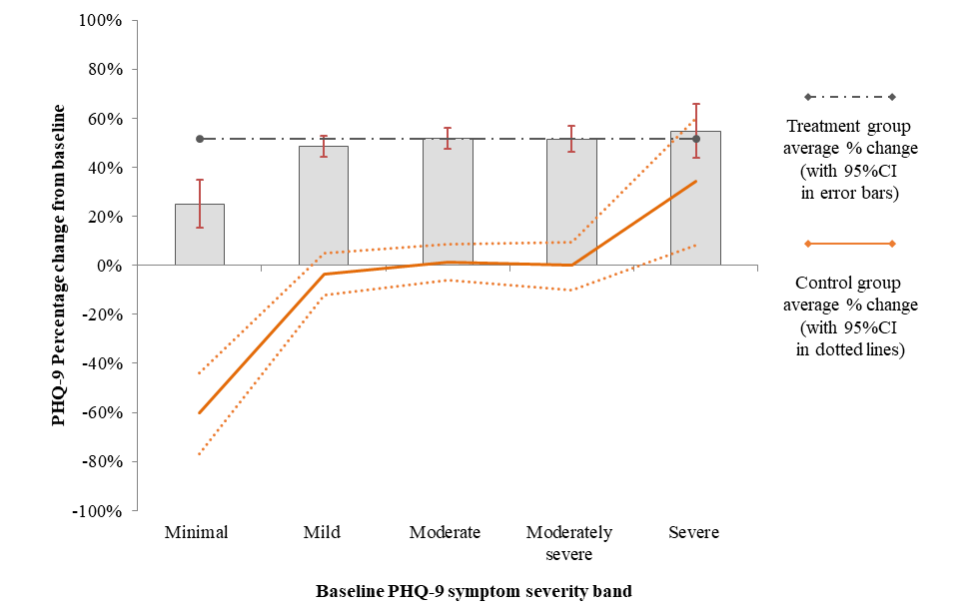
Measurement of mean treatment-related PHQ-9 symptom change as a proportional pattern of remission (52%); per initial pretreatment symptom severity; whiskers represent 95% CIs. Symptom change observed under control conditions indicated by a solid trend line.

In the first step (operationalizing the first hypothesis that individuals with increased symptoms at baseline would also demonstrate increased rates of symptom change), the relationship between baseline symptom severity and the quantity of symptom change was explored graphically. Figure 2, illustrating PHQ-9 change as a linear function, and Figure 3, illustrating PHQ-9 change as a proportional change from baseline, both demonstrate the symptom change on the y-axis within each of the PHQ-9 baseline symptom bands (x-axis). In addition, the symptom change observed within the waitlist condition is included as a dotted trend line, illustrating the trend of nonspecific change in symptoms within each bands of symptom severity at baseline.

Figure 2 illustrates an increased rate of symptom change that was associated closely with increased baseline symptoms. In Figure 2, individuals with severe baseline symptoms were observed to reduce by as much as threefold compared to individuals with mild baseline symptoms (11.4 vs 3.7, respectively). In addition, participants with severe symptoms in the control group demonstrated a sizable reduction in symptoms even when treatment was not applied. This nonspecific symptom-related change was pronounced to the extent that individuals with severe baseline symptoms in the control group demonstrated higher symptom reduction than individuals with moderate symptoms in treatment (7 points vs 6 points, respectively). That is, as a linear effect, the nonspecific symptom change within the control condition was larger than the treatment-related symptom change of individuals with moderate symptoms.

Figure 3 illustrates the proportional percentage change of symptoms within each of the mild, moderate, moderately severe, and severe subgroups. The figure illustrates that as a proportional change, an average treatment-related change of 50% to 55% was observed across all subgroups of individuals who started with at least mild symptoms at baseline. Of note, the rate of proportional improvement in treatment (50%-55%) was greater than the nonspecific change experienced by individuals with severe baseline symptoms in the waitlist conditions (35%). That is, the measurement of change as a percentage change resulted in a clearer differentiation of treatment-specific and nonspecific change.

Table 2 includes the numerical descriptions of change for both the treatment and control conditions. Table 2 also includes effect sizes that were calculated within the treatment group as a whole and the effect size demonstrated by individuals in the mild, moderate, moderately severe, and severe bands of baseline symptoms. Individuals with mild depressive symptoms showed smaller effects (1.59) compared to individuals with more severe symptoms (3.9).

**Figure 4:**
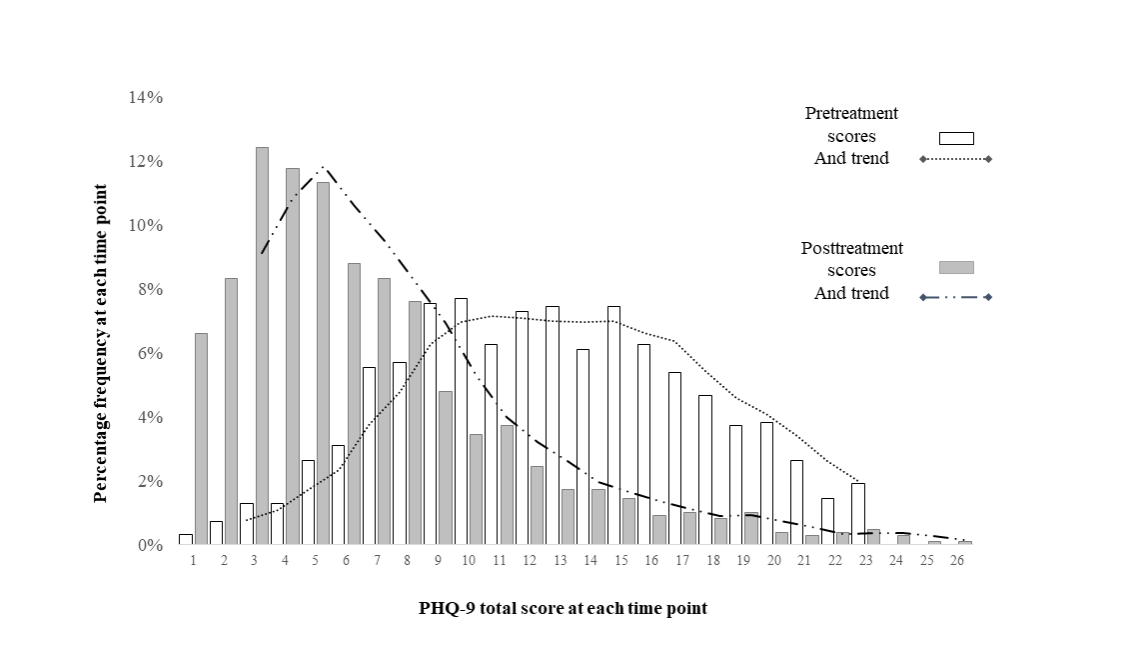
Dispersion of symptom scores (nine-item Patient Health Questionnaire, PHQ-9) at pretreatment (in light bars) and posttreatment scores (in dark bars). The dotted trend lines are indicative of the shape of each distribution.

In a second step, the second hypothesis that distributions of pretreatment and posttreatment depression symptom levels would show evidence of positive skewness and kurtosis was operationalized with an exploration of the distribution of pretreatment and posttreatment symptom scores. Figure 4 illustrates the distribution of PHQ-9 symptom scores, both before and following treatment. These histograms illustrate a slight positive skewness of scores at pretreatment, with fewer individuals presenting within the severely symptomatic band as compared to the mild and moderate bands. In contrast, at posttreatment, increasing positive skewness was observed, where most individuals who reduced their symptoms became concentrated within the mild to minimal symptom ranges. The numerical estimates of the skewness are collated in Table 3.

Taken together, both numerically and graphically, the distributions of symptom scores demonstrated significant positive skewness that increased at posttreatment.

In a third step, the third hypothesis that statistical methods measuring symptom change as a proportional function would be associated with reduced measurement error and indicate greater statistical fit to real symptom data in treatment was operationalized, seeking to explore the model fit of the linear and the multiplicative statistical models of symptom change. Table 4 collates the goodness-of-fit statistics from models that specified either a proportional or linear function of change.

In Table 4, models that specified a proportional function of symptom change demonstrated a several-fold improvement in the model fit statistics within both the GEE and mixed models, including reduced QIC statistics, reduced AIC, and reduced BIC estimates. Table 4 also collated the measurement error associated with the prediction that change occurred as a linear change of six points, or as a percentage improvement (52% reduction from baseline). A notable reduction in the total estimate of PHQ-9 error variance was evident when a proportional function of change was assumed (σ^2^=16.716 vs σ^2^=24.122). This result demonstrated that by characterizing change as a proportional function, the measurement error and remaining unknown individual variation reduced by more than 30%.

**Table 2:** Rates of change of nine-item Patient Health Questionnaire (PHQ-9) scores associated with linear and proportional change functions; estimates per initial baseline symptom subgroups.

PHQ-9 and change functions		Initial symptom severity						Total
		Minimal (n=72)	Mild (n=345)	Moderate (n=381)	Moderately severe (n=244)	Severe (n=56)	Overall sample (treatment) scores	
**Observed PHQ-9, mean (SD)**								
	Pretreatment	2.83 (1.25)	7.32 (1.33)	12.07 (1.40)	16.67 (1.41)	20.86 (0.84)	11.41 (4.79)	
	Posttreatment	2.22 (2.61)	3.71 (3.3)	5.81 (3.92)	8.07 (5.41)	9.45 (4.99)	5.59 (4.57)	
**GEE^a^ (95% CI)^b^**								
	Additive change estimate	0.61 (–0.30 to 1.18)	3.66 (3.30 to 4.02)	6.22 (5.82 to 6.62)	8.66 (7.98 to 9.34)	11.43 (10.14 to 12.73)	6.00 (5.71 to 6.28)	
	Percent proportional change estimate	21% (–1 to 39)	50% (45 to 54)	52% (48 to 55)	52% (48 to 56)	55% (48 to 61)	52 (50 to 54)	
Effect size, Cohen *d* (95% CI)		0.32 (0.01 to 0.63)	1.59 (1.43 to 1.74)	2.34 (2.19 to 2.49)	2.54 (2.33 to 2.74)	3.90 (3.45 to 4.36)	1.27 (1.21 to 1.34)	
**Control group**								
	Change^c^ (95% CI)^b^	–2 (–27 to –1.24)	–0.1 (–0.76 to 0.53)	0.29 (–0.68 to 1.28)	0.48 (–1.01 to 1.15)	7.37 (5.14 to 9.51)	0.68 (–0.37 to 0.16)	
	Percent proportional change estimate, GEE (95% CI)^b^	–61 (–78 to –44)	–4 (–12 to 5)	1 (–6 to 9)	0 (–10 to 10)	34 (8 to 60)	0% (–1 to 1)	

^a^GEE: generalized estimated equation.

^b^Confidence intervals based on modeled marginal means.

^c^Control group change is nonspecific effect.

**Table 3:** Symptom score distributions statistics

Sample and time point		Skewness (SE)	Baseline symptoms, mean (SD)	Effect size, Cohen *d* (95% CI)
**Treatment sample (n=1098)**				1.27 (1.21 to 1.34)
	Pretreatment	0.271 (0.071)^a^	11.73 (4.83)	
	Posttreatment	1.359 (0.076)^a^	5.60 (4.58)	
**Control sample depression (n=96)**				–0.04 (–0.24 to 0.16)
	Pretreatment	0.178 (0.109)	10.91 (4.53)	
	Posttreatment	0.228 (0.109)	11.00 (5.04)	

^a^Statistical significance beyond .05 alpha on a Shapiro-Wilk test for distribution normality; significance is indicative that normal distribution is not supported within the observed sample.

**Table 4:** Model fit statistics and dispersion of model residuals for the treatment sample (n=1098). Model fit criterion was derived from SAS software, version 9.3.

Method of change specified	QIC^a,b^ (GEE^c^ model)	AIC^d,b^ (Mixed)	BIC^e,b^ (Mixed)	Total variance (PHQ-9 σ^2^)
Linear (normal scale)	52457.6	14059.8	14071.3	16.716
Proportional (gamma scale)	2020.5	4041.8	4053.3	24.122

^a^QIC: quasilikelihood under the independence model criterion.

^b^Confidence intervals based on the multiplicative longitudinal GEE model specified in the analytical plan.

^c^GEE: generalized estimated equation.

^d^AIC: Akaike information criterion.

^e^BIC: Bayesian information criterion.

**Figure 5:**
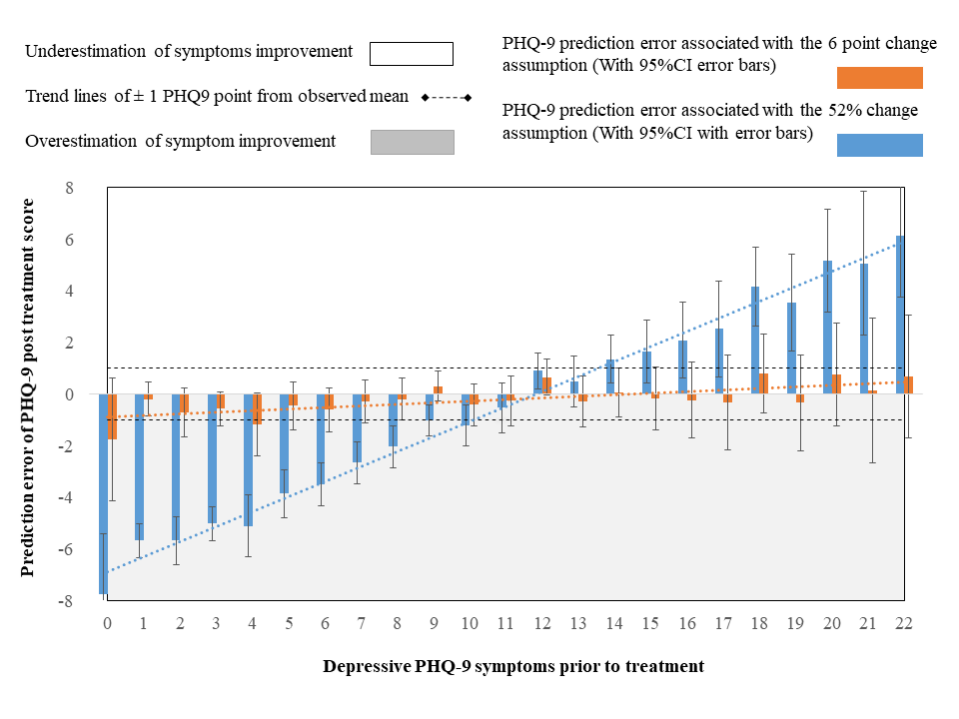
PHQ-9 estimation error (residual) following fixed (linear) and relative (proportional) change assumption.

The measurement error associated with either assumption that change was linear (6 points) or proportional (52%) were graphically explored. Figure 5 illustrates the residual error (y-axis) across individuals who started treatment with different baseline symptoms (x-axis). In the figure, individuals with mild and severe baseline symptoms can be observed to substantially underestimate or overestimate the rate of symptom change when linear change (6 points) was predicted. In contrast, when change was predicted to be proportional (52%), baseline symptoms no longer associated with the rate measurement error. Further, under the proportional assumption, the predicted symptom outcome could be accurately predicted within a single point across individuals with different baselines (marked with dots horizontal lines). In contrast, under the linear assumption, the prediction of symptom outcome become systematically erroneous with baseline severity (a range of up to 16 points between mild and severe).

## Discussion

This study aimed to investigate the statistical characteristic of symptom change in treatment and compare different ways to measure and interpret symptom change. Using a Web-based psychotherapy sample (n=1098), as well as a waitlist control condition (n=96), the statistical characterization of depressive symptom change (PHQ-9) was explored in three steps, corresponding to three proposed hypotheses.

Testing of the first hypothesis demonstrated support for the characterization of symptom change as a proportional function through a clear association between symptom severity at baseline and the rate of change. In contrast, as a proportional estimate of change, individuals in treatment demonstrated a consistent rate of proportional symptom change within all subgroups with mild, moderate, moderately severe, and severe baseline symptom (50%-55%). Critically, the dependency between symptom change and baseline symptom severity was also observed in the waitlist condition, with mild and severe participants changing proportionally in their symptoms even when treatment was not applied. Testing of the second and third hypotheses also illustrated support for the characterization of symptom change as proportional function, with symptom score distributions presenting with positive skewness, particularly following treatment (H2). Similarly, increased model fit, and reduced measurement error was observed when the treatment sample was statistically modeled with an underlying proportional function of change (H3).

The analyses within this study are novel in that they characterize the function of depressive symptom change and compare different statistical methods for measuring and interpreting symptom change within treatment as well as nontreatment conditions. The findings suggest that common psychotherapy symptom scales (eg, PHQ-9) are impacted by a feature of natural bounding at minimal symptoms, which is the suspected culprit for the resulting (1) nonnormal distributions at posttreatment, (2) the dependency between baseline symptoms and rate of change, and (3) the improved model fit for techniques that assume longitudinal change is proportional to baseline.

These findings raise two potentially critical implications for the ability to measure and interpret psychotherapy change in combination with symptom scales. First, the inappropriate use of linear statistics, such as Cohen *d*, when change is proportional would lead to artificially higher estimates of clinical efficacy, both in treatment and in control conditions. For example, in this study, individuals with severe baseline symptoms demonstrated effect sizes that increased by nearly threefold (3.9) when compared to individuals with mild symptoms (1.6), even when the same treatment was applied. This is problematic because linear estimates of change such as Cohen *d* are strongly associated with baseline severity and not with quality or the effectiveness of treatment. This finding is broadly consistent with the data within previous psychotherapy studies showing increased effect sizes with samples of increased symptoms, even when similar treatments are applied [20,29,32].

Second, these findings support a well-established statistical idea posing that the selection of a statistical analysis must match the characteristics of the dataset in order to arrive at valid and accurate statistical measurement, interpretation, and conclusions [4,45]. In this context of depressive symptom scales, the use of proportional statistical analyses resulted in (1) improved statistical modeling of treatment effects, (2) an improved ability to determine what a treatment effect is (50%-55%) and what a nontreatment effect is (35%), as well as for (3) establishing a clinical effect that is robust across individuals with various baseline symptoms (50%-55%). The measurement and interpretation of change as proportional improvement from baseline can also be concretely and easily interpreted as an estimate of change (eg, percentage improvement). Further, in the context of treatment, percentage improvement and percentage change estimates seem to reflect the ideal of treatment (reducing symptoms to minimal) [1,9]. For these reasons, measuring and interpreting change as a fundamentally proportional function can hold critical implications for clinical research that is reliant on accurate and interpretable measurement. For example, researchers seeking to identify clinical moderators, compare between treatments, estimate cost-effectiveness, or classify individual effects are likely to be positively impacted with a suitable choice of analytics that capture the underlying statistical function of change [36,37].

Although the measurement and interpretation of symptom change as a proportional change show promise to increase the accuracy and interpretability of clinical change, several statistical and clinical limitations should be considered about the results of this study. Primarily, the results of this study should be considered as (1) preliminary, (2) specific to a symptom scale of depressive symptoms (PHQ-9), and (3) specific to one kind of treatment model (the Macquarie University online model). Specifically, albeit the strengths of this study as an exploration of change within a large and standardized sample, it is unclear to what extent the 50% to 55% symptom change is specific to this treatment model and to the PHQ-9 scale.

To address these limitations, statistical replication is needed across different symptom scales and treatment models. Specifically, the characterization of symptom change must be observed within other psychotherapy treatment models before more generalizable comments can be made about symptom change and measurement. Future similar studies seeking to characterize and compare symptom change and measurement models could determine to what extent the proportional change pattern generalizes as a measurement principle, across different treatment models and across different symptom scales. In addition, future studies seeking to research this pattern of change could also attempt to compile a meta-analytical characterization of proportional and linear change across different scales and treatment models.

Further, it is important to consider that measurement and interpretation of symptom change as a proportional function is at odds with the widely accepted use of linear statistics in psychotherapy. From one point of view, linear statistics, such as Cohen *d*, are successful as an established measurement standard that can be used to compare change estimates between trials and across clinical instruments [2]. This use of effect sizes has resulted in both enormous amounts of aggregated evidence about the effects of psychotherapy [22] and, for this reason, it is understandable clinical researchers would continue to use this standard for measuring and interpreting symptom change. However, should symptom change occur as a proportional function, the measurement and interpretation of treatment-related change would substantially improve by matching appropriate statistical analysis to the characteristics of the function of symptom change [15,45,46]. A possible solution to this dilemma would be to report both the effect size and percentage estimates of change side by side. In this way, the change that occurs in treatment can be more accurately reported, evaluated, and compared between trials.

Finally, this study does not weigh whether the change rate of 50% to 55% could be evaluated as the same treatment-related effect across individuals with severe or mild baseline symptoms. For example, a symptom reduction demonstrated by individuals with severe baseline symptoms could be interpreted as a more substantive clinical effect than an equivalent symptom reduction achieved with individuals with mild or moderate symptoms [47]. To address these limitations, additional research into the experience of individuals in treatment could determine whether individuals with different baseline symptoms consider the proportional remission pattern an equally satisfactory treatment outcome. For example, Zimmerman and colleagues [48] consider the measurement of patient functionality, positive mental health, and optimism alongside the reduction in depressive symptoms. These additional measures could verify and elaborate on the experience of individuals in treatment and nontreatment conditions, within various symptom bands, shedding more light on the universality or segmentation of the 50% to 55% improvement effect.

In summary, this study aimed to explore the underlying pattern of symptom change and compare different methods for measuring and interpreting depressive symptom change that follows treatment (Web-based psychotherapy). This study has combined evidence of increased rate of change with increased baseline symptoms (hypothesis 1), score distributions that become increasingly skewed following treatment (hypothesis 2), and increased measurement accuracy achieved by statistical methods that assume change is proportional (hypothesis 3) to suggest that the fundamental function of symptom change is proportional. The promise of matching these characteristics of proportional symptom change to a suitable statistical analysis is important for all (1) statistical modeling and the prediction of treatment effects, (2) an improved ability to differentiate treatment and nonspecific symptom change, as well as for (3) determining an estimate of treatment-related change that will not sway with increased baseline symptoms. Replication of these preliminary findings are essential within additional depressive symptom scales, other types of psychological conditions, and across different treatment modalities.

## Abbreviations

AIC: Akaike information criterion
BIC: Bayesian information criterion
GEE: generalized estimated equation
ICBT: Internet-delivered cognitive behavioral therapy
QIC: quasilikelihood under the independence model criterion
